# Function and Benefits of Natural Competence in Cyanobacteria: From Ecology to Targeted Manipulation

**DOI:** 10.3390/life10110249

**Published:** 2020-10-22

**Authors:** Alexandra M. Schirmacher, Sayali S. Hanamghar, Julie A. Z. Zedler

**Affiliations:** Matthias Schleiden Institute for Genetics, Bioinformatics and Molecular Botany, Friedrich Schiller University Jena, 07743 Jena, Germany; alexandra.schirmacher@uni-jena.de (A.M.S.); sayali.hanamghar@uni-jena.de (S.S.H.)

**Keywords:** cyanobacteria, DNA uptake, DNA processing, type IV pili, T4P, *com* genes, *pil* genes, natural competence, transformation, genetic engineering

## Abstract

Natural competence is the ability of a cell to actively take up and incorporate foreign DNA in its own genome. This trait is widespread and ecologically significant within the prokaryotic kingdom. Here we look at natural competence in cyanobacteria, a group of globally distributed oxygenic photosynthetic bacteria. Many cyanobacterial species appear to have the genetic potential to be naturally competent, however, this ability has only been demonstrated in a few species. Reasons for this might be due to a high variety of largely uncharacterised competence inducers and a lack of understanding the ecological context of natural competence in cyanobacteria. To shed light on these questions, we describe what is known about the molecular mechanisms of natural competence in cyanobacteria and analyse how widespread this trait might be based on available genomic datasets. Potential regulators of natural competence and what benefits or drawbacks may derive from taking up foreign DNA are discussed. Overall, many unknowns about natural competence in cyanobacteria remain to be unravelled. A better understanding of underlying mechanisms and how to manipulate these, can aid the implementation of cyanobacteria as sustainable production chassis.

## 1. Introduction

Natural competence refers to the ability of prokaryotes to take up DNA from the environment and insert it into their own genome by homologous recombination. This prokaryotic trait is widespread within many phylogenetic taxa of Proteobacteria, Firmicutes, Chlorobi, Deinococcus-Thermus and Euryarchaeota [1]. The best-studied examples of natural competence are all heterotrophic organisms: *Bacillus subtilis*, *Streptococcus pneumoniae*, *Thermus thermophilus*, *Neisseria gonorrhoeae*, *Vibrio cholerae*, *Helicobacter pylori*, *Acinetobacter* spp. and *Haemophilus influenzae* [1,2]. In all known naturally competent bacteria, except for *Helicobacter pylori* [3,4], the first step of DNA uptake is mediated by type IV pili (T4P). In cyanobacteria, these are also referred to as thick pili in order to distinguish them from morphologically distinct thin pili [5]. T4P are multifunctional cellular appendages known to be involved in natural competence, twitching motility [6], predation [7], cell adhesion [8], biofilm formation [9,10,11], virulence [12] and secretion [13].

Natural competence is not restricted to heterotrophic bacteria, but also present in cyanobacteria. Cyanobacteria are oxygenic photosynthetic prokaryotes found in a wide range of environments, which have also attracted interest as production hosts due to their photoautotrophic growth regime and metabolic versatility. Despite their ecological and biotechnological significance and a large body of literature about these organisms, not much is known about the prevalence of natural competence (first described in [14]). Natural competence offers a simple and efficient method of transformation. Understanding and utilising this trait would therefore also enable faster and easier exploitation of these organisms for applications. Here, we review the molecular mechanisms of DNA uptake and processing, the regulation of natural competence and its prevalence in cyanobacteria. We further discuss the implications of manipulating and exploiting this trait in engineering cyanobacteria.

## 2. The Molecular Basis of Natural Competence

**Figure 1:**
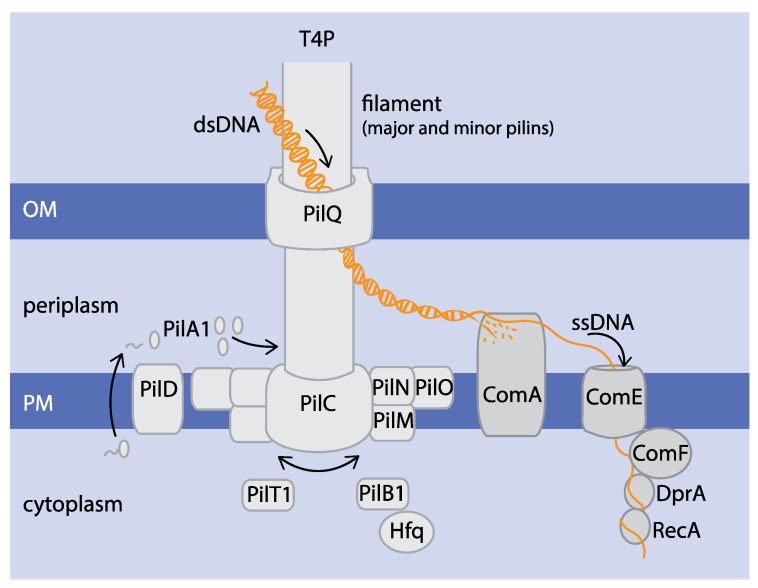
Overview of DNA uptake and processing machinery in *Synechocystis.* Double-stranded DNA (dsDNA) is taken up from the extracellular space through PilQ, the outer membrane (OM) pore of the T4P, by binding to the filament (consisting of major pilin PilA1 and minor pilins). ComA (putative DNA binding and endonuclease domains) is responsible for dsDNA processing into single stranded DNA (ssDNA), which is further translocated across the plasma membrane (PM) via ComE. In the cytoplasm, the ssDNA is further processed by ComF, DprA and RecA.

Transformation via natural competence first involves DNA uptake, followed by DNA processing and homologous recombination. The first step of DNA uptake is mediated by binding to the T4P filament. Upon pilus retraction, the DNA is pulled along with the filament into the periplasmic space and translocated into the cytoplasm [1,15,16]. In cyanobacteria, experimental studies of DNA uptake mechanisms are lacking, however, information regarding the proteins involved is available. Table 1 gives an overview of the proteins and their assigned function in natural competence of cyanobacteria. An overview of how DNA uptake and processing might occur in *Synechocystis* sp. PCC 6803 (hereafter *Synechocystis*) is shown in Figure 1.

### 2.1. T4P Are Crucial for Natural Competence in Cyanobacteria

Cyanobacterial T4P have been shown to play a role in twitching motility [5,29], metal uptake [30,31], biofilm formation [32], flocculation [33] and also in natural competence [14,34]. The filament of T4P mainly consists of the major pilin, which in cyanobacteria, is referred to as PilA1 [5,15,17,35,36]. PilA1 is exported to the periplasm via the General Secretory (Sec) pathway [37]. The peptidase PilD cleaves off the Sec signal peptide and methylates PilA1 [38,39,40]. In addition to PilA1, further minor pilins of low abundance might be incorporated into the filament. In cyanobacteria, these are thought to assist with flocculation [33], motility [41,42] and also seem to play a role in natural competence. For instance, in *Synechocystis* a knockout of the gene encoding for the minor pilin PilA2 (*sll1695*) led to a transformation efficiency of only 52% compared to the wild type (WT) [17], while motility was not affected [5]. In heterotrophic bacteria, minor pilins were shown to have versatile functions such as ensuring correct pilus assembly, assisting in pilus adhesion and, interestingly, they might also be involved in DNA binding [15]. Where exactly the DNA is bound to the filament and if this is mediated by minor or major pilins is not certain. In *V. cholerae*, *Neisseria meningitidis* and *T. thermophilus* minor pilins are thought to be located at the tip of the filament [15,43]. However, other models favour the binding of DNA to the major pilin or to minor pilins along the filament [34]. A similar scenario could also apply to cyanobacteria. For instance, PilA2 and/or other minor pilins in *Synechocystis* could function as DNA binding proteins similarly to PilA3 in *Synechococcus elongatus* PCC 7942 (hereafter *S. elongatus*) [22]. However, this needs experimental validation. Additionally, it has been suggested that the outer membrane pore-forming secretin PilQ, through which the DNA is translocated into the periplasm, may directly be involved in DNA binding [44].

**Figure 2:**
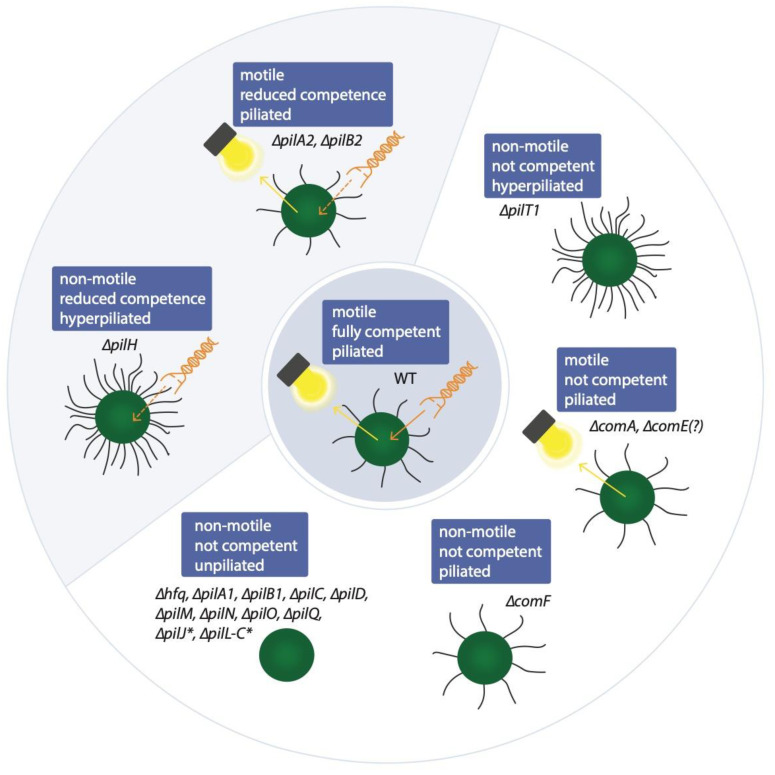
Phenotypes of *Synechocystis* mutants deficient in a single component of the natural competence machinery. Wild type (WT) cells are motile, piliated and competent (centre) while in ∆*pilH*, ∆*pilA2 and* ∆*pilB2* mutants a reduced competence (~30 and 50%) is observed [5,17,26]. Other knockouts lead to strongly limited competence (denoted with an asterisk) or a complete loss of competence [5,17,19,22,23,26].

Further pilus subunits are known to affect natural competence of cyanobacteria, although their function in the T4P system has not been established. For instance, PilB2 seems to have an additional role in natural competence—a Δ*pilB2* mutant shows a reduced transformation efficiency at 37% of the WT while retaining motility [17]. In addition, two gene clusters that encode components of Che-like chemotaxis systems can also indirectly affect natural competence: A *pilH* mutant of *Synechocystis* was shown to accumulate T4P, but lost motility and competence was reduced to 28.6% of WT levels [26]. Similarily, *pilJ* and *pilL-C* mutants were non-motile, non-piliated and competence was reduced to 1.5% of the WT [26]. A *pilI* mutant showed a reduction in the number of T4P, whilst the *pilL-N* mutant was hyperpiliated. PilG does not seem to be involved in competence as a mutant retained transformability [26]. An overview of the different phenotypes of knockout mutants is shown in Figure 2.

A recent study identified several other T4P-related genes as essential for natural competence in *S. elongatus* [22]: *pilA3* (Synpcc7942_2590), *pilW* (Synpcc7942_2591), *rntB* (Synpcc7942_2485), *rntA* (Synpcc7942_2486) and *sigF2* (Synpcc7942_1784). The function of *rntA* and *rntB* is not known, however, interestingly, they contain a type IV pilin-like signal peptide suggesting a role within the T4P. SigF2 is part of the regulatory network inducing natural competence. While *pilA3* and *pilW* are essential for natural competence in *S. elongatus*, these genes are not present in other naturally competent cyanobacteria [22]. Overexpression of *pilA3* also led to a higher transformation efficiency, supporting an essential role of minor pilins for DNA binding.

### 2.2. Com Proteins Mediate DNA Uptake and Processing

Once the DNA has been pulled into the periplasm, one DNA strand is degraded whilst the other is translocated further across the cytoplasmic membrane through the action of ComAEF [1,16,44]. In cyanobacteria, the exact function of each of these proteins is poorly understood. ComA is predicted to be plasma membrane (PM)-localised with periplasmic DNA-binding and endonuclease domains [18]. This protein was shown to be crucial for transformation in *Synechocystis* and dispensable for motility [17]. In *Synechocystis,* ComE has been identified as a homologue to ComEC from *B. subtilis* [17] where it forms a pore in the PM and is proposed to translocate one strand of DNA whilst degrading the other [1,16]. ComF was shown to play a crucial role in both phototaxis and transformation in *Synechocystis* [19]. The *S. pneumoniae* homologue ComFC was proposed to mediate the transition between DNA uptake and homologous recombination. It was shown to interact with DprA and to form a complex with ComFA, an ATPase that also binds to single-stranded DNA (ssDNA) [45]. Translocation of ssDNA across the PM is known to be ComFA-dependent in multiple gram-positive bacteria [1,16] but no homologue has been identified in cyanobacteria. Once the ssDNA reaches the cytoplasm, binding of DprA offers protection from nucleases and mediates the recruitment of the recombinase RecA to the ssDNA. RecA is then responsible for integration into the genome via homologous recombination [1,3,16,21]. Both proteins, DprA and RecA, are vital for homologous recombination. However, these mechanisms remain to be experimentally validated in cyanobacteria.

## 3. Versatile Factors Control Natural Competence

Natural competence in bacteria is generally not a constitutive function. Underlying induction and regulation mechanisms are highly diverse and complex [3,46]. For example, induction of competence can depend on growth phase [1,3,47,48], quorum sensing [47,49,50,51], biofilm formation [50,52,53], nutrient limitation [54], DNA damage [55,56], presence of antibiotics [56,57] or certain substrates [47,58,59,60]. For more detailed insights, the reader is directed to recent reviews [1,3,46,61,62].

In cyanobacteria, one of the competence-inducing factors seems to be the growth phase. *Synechococcus* sp. PCC 7002 and *Synechocystis* were shown to be most competent during the exponential phase, and their transformability is drastically reduced in the stationary phase [63,64]. On the other hand, competence of *S. elongatus* was reported to not depend on the growth phase [65]. Generally, it seems to be species-dependent if and how a specific growth phase influences competence. Despite a correlation of growth phase and level of transformability in several species, the growth phase itself might not be the actual inducer. Many factors, such as nutrient limitation or quorum sensing, correlate with the stationary phase and may thus be the true factor of varied transformability levels. Another trigger of natural competence in cyanobacteria could be changes in lifestyle. For instance, in the plant pathogen *Xylella fastidiosa*, cultivation in microfluidic chambers mimicking its natural environment and promoting biofilm formation was shown to induce competence [52]. Many cyanobacteria are capable of biofilm formation and a link between these factors could exist.

Recently, the circadian clock was identified as a major regulator of natural competence in *S. elongatus* [22]. Cells grown under a light-dark cycle showed the highest transformation efficiency at dusk. Expression of *comEA* is up-regulated at dusk or shortly before, and other genes relevant for competence (*sigF2*, *pilA3*, *rntA* and *dprA)* are induced by darkness. Interestingly, most T4P component genes were expressed in the morning. Consequently, cells were piliated during daytime, and numbers of pili decreased until the second half of the night [22].

There might be many other factors that impact natural competence in cyanobacteria, but these might be difficult to identify. For instance, heterocyst-forming species lead a complex lifestyle and excrete a large number of extracellular nucleases [66], making the characterisation of their competence systems difficult. Many filamentous cyanobacteria produce hormogonia, which are known to produce T4P and to be motile [67]. The hormogonial state might be more suitable for the uptake and incorporation of exogenous DNA than the filamentous state as the new genetic information could be transferred during cell division. Thus, factors influencing and regulating natural competence in cyanobacteria are diverse and complex. It is likely that many more remain to be unravelled.

## 4. Natural Competence Might Be More Frequent among Cyanobacteria than Initially Anticipated

### 4.1. Experimental Evidence of Natural Competence in Cyanobacteria

The first record of natural competence in cyanobacteria is from 1970 for *Synechococcus* sp. PCC 7943 (previously *Anacystis nidulans* 602) [14]. Over the years, multiple members of the *Synechococcus* genus were found to be naturally competent: *S. elongatus* R2 [68], *S. elongatus* PCC 11801 [69] and *Synechococcus* sp. PCC 7002 [70]. It is unclear if *Synechococcus* sp. PCC 6301 is in fact naturally competent as there are many older records reporting it as such [63,71,72,73,74,75], although other studies claim the opposite [76,77]. This strain might have lost this trait over decades of lab cultivation or its competence machinery might be controlled by an unknown inducer. Most records of natural competence in cyanobacteria are found for the model organism *Synechocystis*, where it was first described in 1982 [33]. Apart from this, only few other, mostly unicellular, cyanobacteria are known to be naturally competent, including *Thermosynechococcus elongatus* BP-1 [78] and *Microcystis aeruginosa* PCC 7806 [79].

Records of natural competence in filamentous cyanobacteria are very scarce, suggesting it might not be common within this morphological group. In fact, filamentous cyanobacteria are widely regarded as being not naturally competent [80,81,82]. One case of natural competence was recorded for *Nostoc muscorum* in 1981 [83], and two more records of natural competence in this species exist from 1987 [84] and 1990 [85]. The latter study also reported the successful transformation of the filamentous *Anabaena doliolum* [85]. Since then, no more records of natural competence of *N. muscorum* or *A. doliolum* exist. After several decades without progress, two recent studies have demonstrated successful natural transformation in the filamentous *Phormidium lacuna* HE10DO [86] and the ramified *Chlorogloeopsis fritschii* PCC 6912 [87]. These recent findings may prompt investigations into other filamentous cyanobacteria and indicate that natural competence might be more common in this morphological group than the few literature records denote.

### 4.2. Genomics Give Insights into the Prevalence of Cyanobacterial Natural Competence

A bigger picture of how widespread natural competence is among cyanobacteria is still lacking. However, recent studies suggest a higher prevalence than experimental evidence has so far shown [22,86]. A total of 345 cyanobacterial genomes were recently analysed for their presence of the genes *pilA1*, *pilD*, *pilB*, *pilT*, *pilC*, *pilM*, *pilN*, *pilO* and *pilQ* and combined with data on *comA*, *comE* and *comF* from a preceding study [88,89]. However, *com* gene data are only available for 21% of the 345 again analysed species. Therefore, after curating this dataset by retaining only species where data for both *pil* and *com* genes were available, this resulted in 73 cyanobacterial genomes (Table S1, taxonomy retrieved from NCBI taxonomy browser, accessed 12 September 2020). An overview of the presence of *pil* genes in our cyanobacterial database is given in Table 2.

These data show that 63% of the analysed species have at least one copy of each of the *pil* and *com* genes. This suggests that the majority of cyanobacteria have a full complement of T4P genes. 46 out of 48 of all species which have a full set of *pil* genes, also have a full set of *com* genes (Table S1). Conversely, among the 61 species which have all *com* genes, 15 lack at least one *pil* gene. The dataset contains two members of the basal order of Gloeobacterales: *Gloeobacter violaceus* PCC 7421 and *Gloeobacter kilaueensis* JS1. Both species contain all three *com* homologues and a full (*G. violaceus*) or almost full (*G. kilaueensis*) set of *pil* genes. The presence of these genes in the *Gloeobacter* lineage, that diverged early from all other cyanobacterial lineages [90], suggests an early emergence of these genes in cyanobacteria. Thus, natural competence might be a primary trait in cyanobacteria but was lost during evolution in some lineages.

Interestingly, a large proportion of cyanobacterial species lacking *pil* or *com* genes belong to the genus *Prochlorococcus* (Table S1). These are marine, free-living picocyanobacteria, typically with minimal genomes, abundant in subtropical and oligotrophic oceans [91]. Considering their unique characteristics, it is not surprising that members of this genus also lack the *pil* and *com* genes. However, there are a few exceptions: *Prochlorococcus marinus* MIT 9303 and MIT 9313, which both have all of the analysed genes apart from *pilM.* These two strains are very distinct from ‘typical’ *Prochlorococcus* strains as they have a larger cell and genome size and are the only *Prochlorococcus* species known to possess *dprA* [91] and an *hfq* homologue [24]. To the best of our knowledge, it has not been experimentally shown whether these strains are naturally competent.

The list of potentially naturally competent cyanobacteria might even be longer than suggested by this dataset due to the limited information available on the *com* genes. However, it becomes apparent that, especially in filamentous cyanobacteria, natural competence might be more prevalent than so far acknowledged.

## 5. Benefits and Drawbacks of Natural Competence in an Ecological Context

What benefits are derived for cyanobacteria from the uptake and integration of external DNA into their genome? Or, in other words, what are the evolutionary and ecological benefits of natural competence? The two most discussed hypotheses regarding the importance of competence are DNA-for-food and DNA-for-diversity (reviewed in [1,61]). In short, the DNA-for-food hypothesis favours the idea that competent bacteria take up foreign DNA as a nutrient, whilst the DNA-for-diversity explains the benefit of natural competence in acquiring new traits. It has been shown that *Synechocystis* can efficiently utilise external genomic DNA as a phosphate source and also as a carbon source under heterotrophic growth conditions [92], supporting the DNA-for-food hypothesis in cyanobacteria. Evidence can often be interpreted in favour of either of the hypotheses. For example, the induction of competence under nutrient-limiting conditions in some bacteria [1] could be directly linked to nutrient acquisition. Simultaneously, induction of natural competence as a stress response could also increase chances of acquiring new traits that may result in a selective advantage.

Cyanobacteria are present in a wide range of ecological niches and environments, including extreme habitats such as deserts and hot springs. Heterotrophic thermophilic bacteria, e.g., *Thermus thermophilus*, were shown to have high transformation efficiencies [1,93,94,95]. The same may likely apply to thermophilic cyanobacteria inhabiting such extreme habitats. In these environments natural competence can offer swift adaptation strategies in line with the DNA-for-diversity hypothesis.

Natural competence likely offers further ecological advantages beyond these more established hypotheses. For instance, it has previously been brought in context with sexual selection in bacteria, which is in line with the DNA-for-diversity hypothesis [96]. Under conditions unfavourable for replication, horizontal gene transfer may be the favoured way of transferring genetic material—similar to the concept of horizontal gene transfer promoting genetic mixing within a population [97]. Some bacteria secrete DNA into the environment, which is also the case for cyanobacteria [98]. No matter if environmental DNA stems from cell lysis or secretion, natural competence (as a mean of horizontal gene transfer) could promote genetic diversity, to some extent, analogous to sexual reproduction in animals.

Natural competence as a trait does not only offer benefits but also entails significant costs and potential drawbacks from an ecological point of view. Expressing the molecular machinery required for natural competence is costly. The cost of T4P formation can, however, be offset to some extent, given its functions across a wide range of important cellular mechanisms [34,35,44]. Sharing the cost for T4P formation means that the cost for natural competence itself is significantly reduced to mainly the Com proteins. Another risk comes with the uptake and potential incorporation of defective or harmful genes [1]. However, polyploidy is widespread amongst cyanobacteria (reviewed in [99]) and might efficiently compensate for this risk. Many cyanobacteria also have efficient restriction-modification systems that degrade foreign DNA without a matching methylation pattern [100], which is likely to offer additional protection. Additionally, CRISPR-Cas (clustered regularly interspaced short palindromic repeats/CRISPR associated proteins) systems are widespread amongst cyanobacteria (reviewed in [101,102]) and could also play a role in cellular protection from foreign DNA. Overall, this suggests that the benefits of natural competence, at least for cyanobacteria, clearly prevail the drawbacks.

## 6. Targeted Manipulation of Natural Competence

Cyanobacteria are of interest not only from a fundamental point of view given their immense ecological importance but also for applications as they are considered promising chassis to establish sustainable, light-driven biotechnological processes for a plethora of products from commodities to high-value compounds [103,104,105,106]. An important prerequisite for developing a target strain is the availability of reliable genetic manipulation tools and efficient transformation methods. Amongst the DNA delivery methods available for cyanobacteria, natural competence constitutes a simple and effective way of delivering DNA into a strain of interest. Thus, understanding underlying mechanisms and regulation of natural competence can open up new avenues of targeted manipulation and wider exploitation of these organisms.

**Figure 3:**
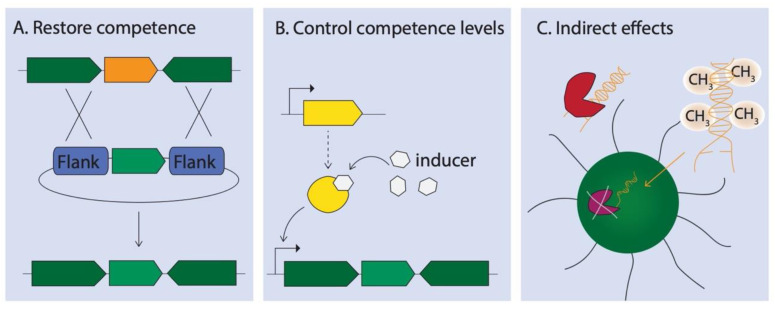
Examples of strategies for manipulating natural competence. (**A**) Restoration of natural competence by exchanging a non-functional gene (shown in orange) with a functional copy, e.g., by homologous recombination. (**B**) Controlling competence with a known (engineered) regulator, e.g., an external inducer molecule that binds to a transcription factor that activates a promoter for transcription of competence genes. (**C**) Control indirect effects on transformability by, e.g., knocking out nucleases (intracellular or excreted) or methylating DNA for transformation.

Many cyanobacterial species contain all or almost all genes required for natural competence (Table 2 and Table S1), however experimental evidence of their transformability is still largely lacking. Restoring natural competence of some cyanobacterial species might be possible by replacing non-functional genes or adding functional copies from related cyanobacteria (Figure 3A). For instance, the fast-growing strain *Synechococcus elongatus* UTEX 2973 was found to not be naturally competent [107]. This is, at least partly, due to a mutation in the *pilN* gene [108]. Introducing a second, functional copy of *pilN* from *S. elongatus* into a neutral site on the genome lead to a restoration of natural competence [108]. However, the authors suggest the level of natural competence is lower than in *S. elongatus* [108]. Similar strategies could also be employed in other strains. It is important to note that restoring the target gene at its native locus might be beneficial to conserve regulatory elements and expression levels with other proteins of the competence machinery. In *Pseudomonas aeruginosa*, the stoichiometry of the PilM, PilN and PilO proteins was found to be important for stable PilM/N/O complex formation [109]. This is likely to also be the case in cyanobacteria, therefore introducing *pilN* into its native locus in *S. elongatus* UTEX 2973 might lead to an improved transformation efficiency.

Another interesting approach is to use on/off switches or even titratable systems, using defined external cues, to tightly control levels of natural competence (Figure 3B). Several inducible promoter systems have been characterised in cyanobacteria [110,111,112] and multiple types of genome editing tools have been developed (recently reviewed in [113]). These tools could also be deployed to engineer natural competence. The use of inducible systems with known effector molecules could potentially circumvent the need to depend on native (often unknown) regulators of natural competence.

Difficulties with genetic manipulation by natural competence might, however, not only stem from a lack or disruption of the machinery itself but might be due to indirect effects. Many cyanobacteria, particularly filamentous species [82], have endogenous restriction-modification (RM) systems to protect the cell from incoming foreign DNA [100]. RM systems consist of methyltransferases, which methylate own DNA, and restriction endonucleases that digest DNA lacking the matching methylation pattern. However, restriction endonucleases only cut double-stranded DNA and should, therefore, not affect ssDNA as taken up by natural competence [96]. Nevertheless, DNA degradation by cellular protection mechanisms and extracellular nucleases might indirectly contribute to the lack of transformability in some cyanobacterial species. In *Synechocystis*, the deactivation of the exonuclease RecJ was shown to dramatically improve transformation efficiency [114]. Similarly, deletion of a type I restriction endonuclease in *T. elongatus* BP-1 also leads to higher transformation efficiencies [115]. Furthermore, pre-methylation of DNA for transformation could aid in overcoming transformability issues [116,117] (Figure 3C).

Ultimately, further research is needed to obtain a better understanding of natural competence in cyanobacteria. The development of strategies for controlling and manipulating natural competence is now enabled by advances in genetic engineering and genome editing tool development in cyanobacteria. This can not only contribute to understanding the molecular mechanisms of natural competence and its ecological context but also allows the wider exploitation of these organisms biotechnologically.

## Figures and Tables

**Table 1:** Proteins known to be involved in natural competence of *Synechocystis*.

Protein	Gene Assignment	Function	Reference
ComA	*slr0197*	DNA translocation (DNA binding and nuclease activity?)	[17,18]
ComE	*sll1929*	DNA translocation (translocase activity?)	[17]
ComF	*slr0388*	DNA translocation (transitioning DNA uptake and homologous recombination?)	[19]
DprA	*slr1197*	DNA processing protein	[20,21,22]
Hfq	*ssr3341*	Pilus biogenesis	[23,24]
PilA1	*sll1694*	Filament formation (major pilin)	[5]
PilA2	*sll1695*	Filament formation (minor pilin)	[5,17]
PilB1	*slr0063*	Motor protein (polymerisation)	[17]
PilB2	*slr0079*	Unknown	[17]
PilC	*slr0162-slr0163*	Platform protein	[5]
PilD	*slr1120*	Prepilin peptidase	[5,17,25]
PilH	*slr1042*	Che-like response regulator pilus assembly	[26]
PilJ	*slr1044*	Che-like response regulator pilus assembly	[26]
PilL-C	*slr0322*	Che-like response regulator pilus assembly	[26]
PilM	*slr1274*	Pilus alignment complex	[17]
PilN	*slr1275*	Pilus alignment complex	[17]
PilO	*slr1276*	Pilus alignment complex	[17]
PilQ	*slr1277*	Secretin	[17]
PilT1	*slr0161*	Motor protein (depolymerisation)	[5,27]
RecA	*sll0569*	Homologous recombination	[20,21,28]

**Table 2:** Identification of *pil (pilA1, pilD, pilB, pilT, pilC, pilM, pilN, pilO, pilQ)* and *com (comA, comE, comF)* genes across cyanobacterial orders. Cells are highlighted in shades of green correlating to their number.

Order	Number of Species	Number of *pil* and *com* Genes Identified in the Genome												
		0	1	2	3	4	5	6	7	8	9	10	11	12
Chroococcales	10	0	0	0	0	0	0	0	0	0	0	0	2	8
Chroococcidiopsidales	1	0	0	0	0	0	0	0	0	0	0	0	0	1
Gloeobacterales	2	0	0	0	0	0	0	0	0	0	0	0	1	1
Nostocales	13	0	0	0	0	0	0	0	0	0	0	1	0	12
Oscillatoriales	8	0	0	0	0	0	0	0	0	0	0	0	1	7
Pleurocapsales	2	0	0	0	0	0	0	0	0	0	0	0	0	2
Synechococcales	37	2	3	0	1	1	2	0	2	1	0	2	8	15

## Supplementary Materials

The following are available online at https://www.mdpi.com/2075-1729/10/11/249/s1, Table S1: Prevalence of *pil* and *com* genes within cyanobacterial species.
